# Downregulation of KIAA1199 alleviated the activation, proliferation, and migration of hepatic stellate cells by the inhibition of epithelial–mesenchymal transition

**DOI:** 10.1515/med-2023-0689

**Published:** 2023-04-06

**Authors:** Jingmei Liu, Suhong Xia, Ping Han, Mingyu Zhang, Jingwen Wu, Jiazhi Liao

**Affiliations:** Department of Gastroenterology, Tongji Hospital of Tongji Medical College, Huazhong University of Science and Technology, Wuhan, Hubei Province, China; Department of Gastroenterology, Hainan Hospital of PLA General Hospital, Sanya, Hainan, China

**Keywords:** KIAA1199, hepatic stellate cells, liver fibrosis

## Abstract

KIAA1199, a major glycosaminoglycan component of the extracellular matrix, was reported to induce a fibrosis-like process. However, the relationship between KIAA1199 and liver fibrosis remains unclear. The liver fibrosis mouse model was established with carbon tetrachloride (CCl_4_). Here, we found that KIAA1199 was upregulated in CCl_4_-induced liver fibrosis. The expression of KIAA1199 was also increased in TGF-β-stimulated LX-2 cells. To clarify the impact of KIAA1199 in hepatic stellate cells (HSCs), we downregulated the expression of KIAA1199 in LX-2 cells by RNA interference. Cell proliferation, apoptosis, and migration were determined by CCK-8, flow cytometry, and transwell assay. We found that KIAA1199 knockdown reduced the expression of fibrosis markers α-SMA and COL1A1. Depletion of KIAA1199 inhibited cell proliferation by downregulating cyclin B1 and cyclin D1 and promoted cell apoptosis by upregulating Bax and downregulating Bcl-2. Moreover, KIAA1199 knockdown decreased matrix metalloproteinase-2 (MMP-2) and MMP-9 expression to inhibit the migration ability of LX-2 cells. Silencing KIAA1199 also suppressed the epithelial–mesenchymal transition phenomenon. Collectively, our study revealed that KIAA1199 knockdown alleviated the activation, proliferation, and migration of HSCs, while promoting apoptosis of HSCs, which suggests that KIAA1199 may be a potential regulator of liver fibrosis.

## 1 Introduction

Liver fibrosis is a common pathological alteration under persistent liver injury, which may eventually lead to cirrhosis or even liver cancer [1]. The excessive deposition of the extracellular matrix (ECM) is a typical characteristic of liver fibrosis [2]. Hepatic stellate cells (HSCs), which are “quiescent” in the normal liver, become “activated” after liver injury, acquire a myofibroblast phenotype, and result in the accumulation of ECM [3]. Understanding the mechanism of HSC activation is of great value for the development of novel strategies that prevent the progression of liver fibrosis.

KIAA1199, also known as cell migration inducing protein (CEMIP) and hyaluronan binding protein (HYBID), is an important member of the KIAA family in the Human Unidentified Gene-Encoded (HUGE) database [4]. KIAA1199 was first identified as an inner ear-specific protein in 2003 [5]. Subsequently, the increased expression of KIAA1199 was detected in many tumor tissues and strongly related to tumorigenesis and poor prognosis [6,7,8]. Moreover, gain- and loss-of-function studies demonstrated a potential biological role of KIAA1199 in the regulation of cell proliferation, invasion, and migration [8,9]. Further studies demonstrated that KIAA1199 may be a regulator of the epithelial–mesenchymal transition (EMT). KIAA1199 silencing induced the expression of epithelial markers (E-cadherin and claudins) and decreased the expression of mesenchymal markers (N-cadherin, vimentin, and snail1) [10,11]. EMT plays an important role in liver fibrosis, and abnormal activated EMT promotes the activation and migration of HSCs, thus contributing to the deposition of ECM in the liver [12,13]. Recently, KIAA1199 was reported to induce a fibrosis-like process in osteoarthritic chondrocytes [14]. However, the role of KIAA1199 in liver fibrosis has not been investigated so far.

In this study, we found that the expression of KIAA1199 was increased in the CCl_4_-induced liver fibrosis mouse model and TGF-β-stimulated LX-2 cells. Depletion of KIAA1199 inhibited activation, proliferation, and migration, while promoting apoptosis of HSCs. Further studies revealed that KIAA1199 knockdown also suppressed EMT. These findings unveil a novel role of KIAA1199 in liver fibrosis.

## 2 Materials and methods

### 2.1 Establishment of a liver fibrosis mouse model

Male C57BL/6 wild‐type (WT) mice, aged 6–8 weeks, were used in this study. Mice were divided into the following three groups: (i) Sham (*n* = 6), (ii) CCl_4_ for 4 weeks (*n* = 6), and (iii) CCl_4_ for 8 weeks (*n* = 6). Mice in the CCl_4_ group were intraperitoneally injected with CCl_4_ (1:20 dissolved in corn oil) twice a week. WT mice that received an equal volume of corn oil at the same time point served as controls. After 8 weeks, mice were killed and liver tissues were isolated for subsequent experiments. All animal studies were approved by the Institution Ethics Committee of Tongji Medical College, Huazhong University of Science and Technology.

### 2.2 Immunohistochemistry

Mouse liver tissue samples were fixed with 4% paraformaldehyde at room temperature for 48 h, embedded in paraffin, and cut into 5 µm thick sections. The 5 µm thick sections were used for hematoxylin and eosin (H&E), Masson and Sirius red stain. KIAA1199 (21129-1-AP, Proteintech) and α-SMA antibodies (Ab124961, Abcam) were used to detect the expression of KIAA1199 and α-SMA in mouse fibrotic liver tissues.

### 2.3 Cell culture and transfection

The human hepatic stellate cell line LX-2 was acquired from the Institute of Liver and Gastrointestinal Diseases, Huazhong University of Science and Technology, and cultured in DMEM with 10% FBS (Gibco, Invitrogen, USA). For transfection, small interfering RNA (siRNA) against KIAA1199 (KIAA1199-siRNA) and control siRNA were synthesized by RiboBio (Guangzhou, China). KIAA1199 siRNA sequences were as follows: GATCCTTACTATGGTCTGA and GGAGTGGTTCGATCATGAT.

The siRNAs were transfected into cells using Lipofectamine^®^ 3000 (Invitrogen, Carlsbad, USA) according to the manufacturer’s instructions.

### 2.4 Quantitative real‐time PCR

Total RNA was extracted using the TRIzol reagent (Invitrogen, CA, USA), and cDNA was synthesized using the PrimeScript RT reagent kit (Abclonal, China). The real-time PCR was performed using SYBR Premix ExTaq (Abclonal, China) on an ABI QuantaStudio Real-Time System (Applied Biosystems, Carlsbad, CA, USA). The sequences of the primers are listed in Table 1.

**Table 1:** Primer sequences for PCR

Gene	Forward (5′-3′)	Reverse (3′-5′)
mKIAA1199	TGATGGGAGTCGAGGTCAC	GAGCACTATGGAATTGTCAGGG
mα-SMA	GCGTGGCTATTCCTTCGTGACTAC	CGTCAGGCAGTTCGTAGCTCTTC
mβ-actin	TGCTGTCCCTGTATGCCTCTG	TGATGTCACGCACGATTTCC
hKIAA1199	CACGGTCTATTCCATCCACATC	GGTTCGCAAAACAATCGGCT
hα-SMA	AAAAGACAGCTACGTGGGTGA	GCCATGTTCTATCGGGTACTTC
hCOL1A1	GAGGGCCAAGACGAAGACATC	CAGATCACGTCATCGCACAAC
hCCNB1	AATAAGGCGAAGATCAACATGGC	TTTGTTACCAATGTCCCCAAGAG
hBax	CCCGAGAGGTCTTTTTCCGAG	CCAGCCCATGATGGTTCTGAT
hBcl-2	GGTGGGGTCATGTGTGTGG	CGGTTCAGGTACTCAGTCATCC
hCCND1	GCTGCGAAGTGGAAACCATC	CCTCCTTCTGCACACATTTGAA
hMMP2	CCAGATGTGGCCAACTACAA	GGTCAGGTGTGTAACCAATGA
hMMP9	CAGTACCGAGAGAAAGCCTATT	CAGGATGTCATAGGTCACGTAG
hE-cadherin	TGATGAGGAAGGCGGTGGAGAAG	CGGTCGAGGTCTGTACTGAGGTG
hN-cadherin	CGATAAGGATCAACCCCATACA	TTCAAAGTCGATTGGTTTGACC
hSnai11	CCTCGCTGCCAATGCTCATCTG	GCTCTGCCACCCTGGGACTC
hVimentin	TGAATGACCGCTTCGCCAACTAC	CTCCCGCATCTCCTCCTCGTAG
hClaudin-1	TCTTGCAGGTCTGGCTATTTTA	TTGGGTAAGAGGTTGTTTTTCG
hβ-actin	CATGTACGTTGCTATCCAGGC	CTCCTTAATGTCACGCACGAT

### 2.5 Western blot

The total protein of cells was extracted with cell lysis buffer (Pierce, Rockford, IL, USA). Cell lysates were separated by 10% SDS-PAGE and then transferred to a polyvinylidene fluoride membrane (Millipore, Bedford, MA, USA). The membrane was blocked with 5% non-fat milk for 2 h and incubated with corresponding antibodies overnight at 4°C. The primary antibodies used were as follows: α-SMA (1:10,000, ab124964; Abcam), KIAA1199 (1:1,000, 21129-1-AP; Proteintech), COL1A1 (1:1,000, 67288-1-Ig; Proteintech), cyclin B1 (1:1,000, 12231; CST), cyclinD1 (1:1,000, 2978; CST), Bax (1:1,000, 5023; CST), Bcl-2 (1:1,000, 4223; CST), E-cadherin (1:1,000, 20874-1-AP; Proteintech), N-cadherin (1:1,000, 22018-1-AP; Proteintech), claudin-1 (1:1,000, A2196; Abclonal), Snai1 (1:1,000, 13099-1-AP; Proteintech), vimentin (1:1,000, 10366-1-AP; Proteintech), MMP2 (1:1,000, 4022; CST), MMP9 (1:1,000, 3852; CST), and β-actin (1:5,000, 66009-1-AP; Proteintech). The membranes were incubated with diluted secondary antibody for 2 h. Protein bands were visualized using an ECL kit (Pierce). Image J software was used to quantify the results.

### 2.6 Cell proliferation assay

Cells were seeded in a 96-well plate at 3,000 cells per well. Cell proliferation was evaluated using the Cell Counting Kit-8 (CCK-8; Dojindo Molecular Technologies, Tokyo, Japan) according to the manufacturer’s protocols.

### 2.7 Cell apoptosis analysis

The Annexin V-FITC apoptosis kit was purchased from BD Pharmingen (San Diego, CA, USA). Cell apoptosis analysis was performed using a FACS Calibur flow cytometer (Becton Dickinson, San Diego, CA) according to the manufacturer’s protocol. Briefly, LX-2 cells were washed twice with PBS and then resuspended in 200 µL of the binding buffer. Then, they were stained with 5 µL of Annexin V for 5 min and 5 µL of propidium iodide (PI) for 10 min in the dark at 37°C. Later, cells were examined using flow cytometry.

### 2.8 Transwell assay

Briefly, 3 × 10^4^ LX-2 cells were resuspended in the serum-free DMEM and plated in the upper chamber, and the lower chamber was inoculated with the DMEM containing 10% FBS. After 24 h, migrated cells were fixed with 4% paraformaldehyde, stained with crystal violet, and then photographed under a light microscope.

### 2.9 Statistical analysis

All statistical analysis was performed using GraphPad Prism 8.0. Data are presented as mean ± standard deviation. Student’s *t*-test was performed to assess the significance of differences between the two groups. *P*-value ＜0.05 was considered statistically significant.


**Ethical approval:** The research related to animal use has complied with all the relevant national regulations and institutional policies for the care and use of animals.

## 3 Results

### 3.1 KIAA1199 was upregulated in the liver fibrosis mouse model

**Figure 1:**
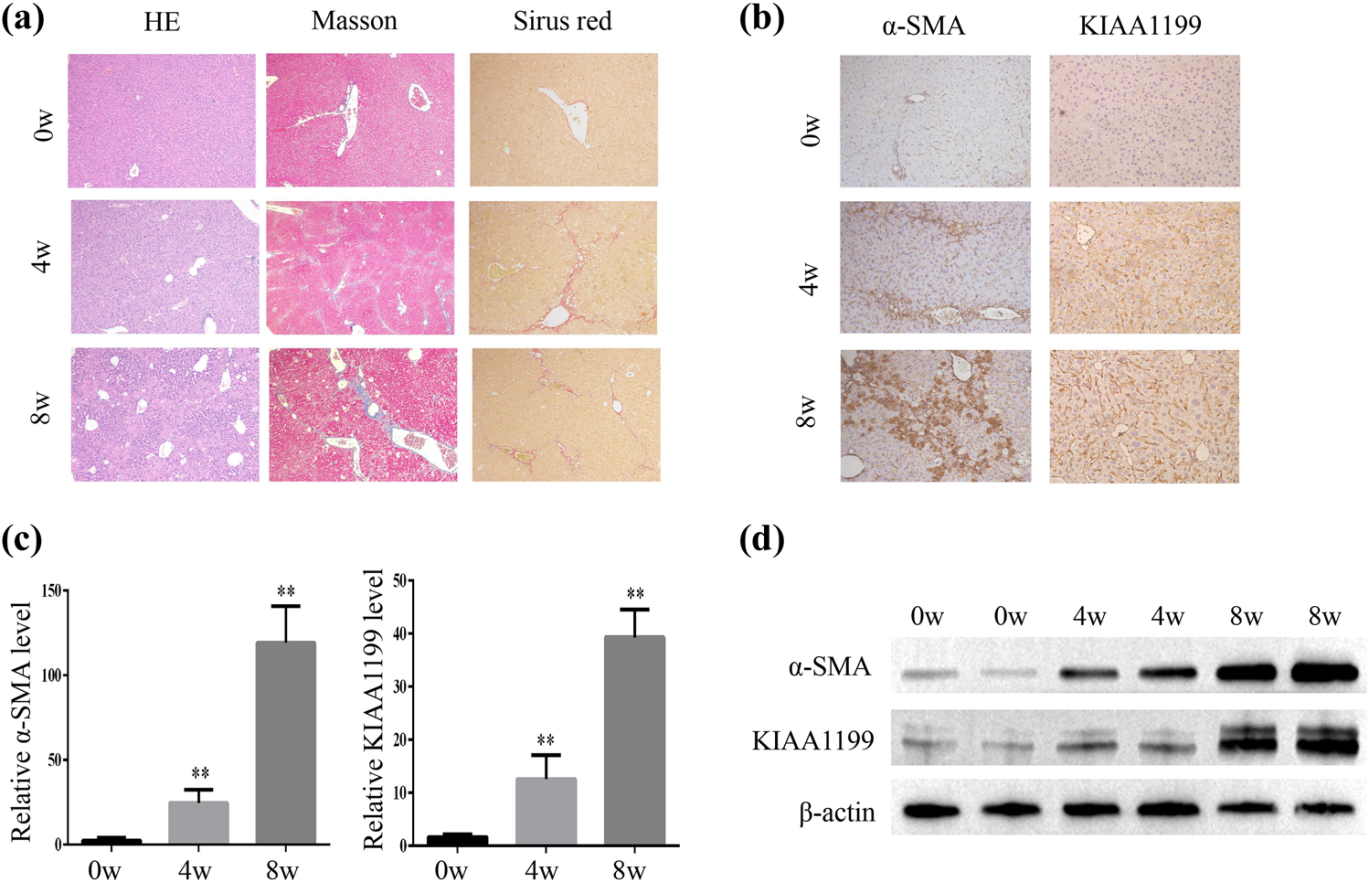
KIAA1199 was upregulated in the liver fibrosis mouse model. (a) Representative H&E, Masson and Sirius Red staining (100×) of the CCl_4_-induced liver fibrosis mouse model. (b) Immunohistochemistry (100×) for α-SMA and KIAA1199 in the liver fibrosis mouse model. (c) The mRNA levels of α-SMA and KIAA1199 in the liver fibrosis mouse model. (d) Representative image showing the protein levels of α-SMA and KIAA1199 in the liver fibrosis mouse model. β-Actin was used as a loading control. Each bar represents mean ± standard deviation of three separate experiments. ^**^ *P* < 0.01.

To explore the role of KIAA1199 in the development of liver fibrosis, we first successfully established a mouse model of liver fibrosis with CCl_4_ (Figure 1a). Immunohistochemistry showed that KIAA1199 and α-SMA were upregulated in mouse fibrotic liver tissues (Figure 1b). Similarly, PCR and western blot analysis also revealed that the KIAA1199 level was significantly increased in mouse fibrotic liver tissues (Figure 1c and d). Moreover, the expression of α-SMA, a marker of HSCs activation, was significantly higher in fibrotic liver tissues compared to the control liver tissues (Figure 1c and d). Collectively, our results indicated that KIAA1199 participates in the development of liver fibrosis.

### 3.2 KIAA1199 was involved in the activation of HSCs

**Figure 2:**
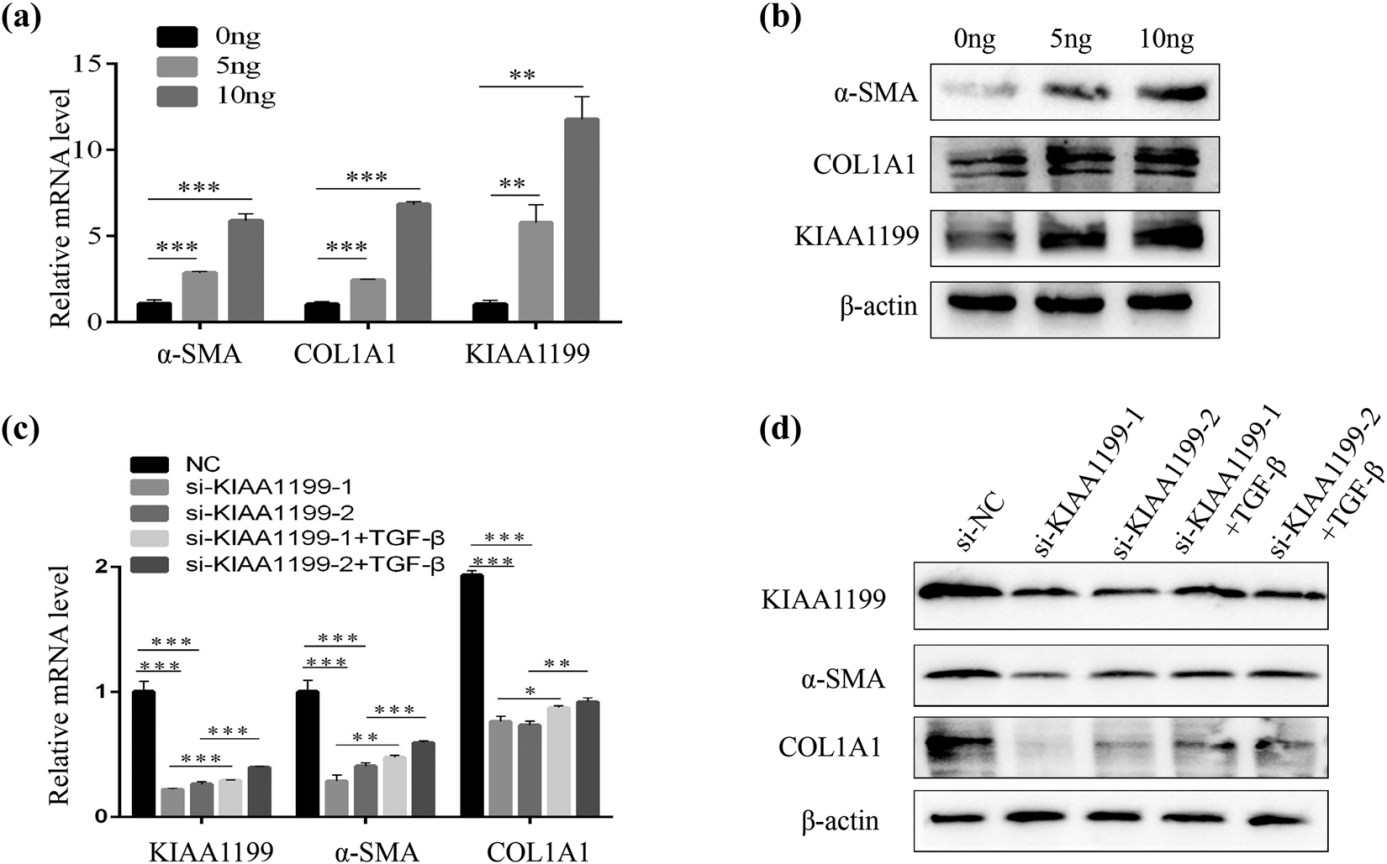
KIAA1199 was involved in the activation of HSCs. (a) The mRNA levels of α-SMA, COL1A1, and KIAA1199 in LX-2 cells after TGF-β stimulation. (b) The protein levels of α-SMA, COL1A1, and KIAA1199 in LX-2 cells after TGF-β stimulation. (c) RT-qPCR and (d) western blot analysis of α-SMA, COL1A1, and KIAA1199 expression in LX-2 cells transfected with siRNAs (si NC or si KIAA1199), pretreated with or without TGF-β. β-Actin was used as a loading control. Each bar represents mean ± standard deviation of three separate experiments.^**^ *P* < 0.01 and ^***^ *P* < 0.001.

To further explore the role of KIAA1199 in the activation of HSCs, we first treated LX-2 cells with different concentrations of TGF-β, a key cytokine in HSC activation [15]. As expected, the expression of fibrosis markers α-SMA and type 1 collagen (COL1A1) were elevated in a dose-dependent manner (Figure 2a and b). Interestingly, KIAA1199 mRNA and protein levels were also upregulated in TGF-β-stimulated LX-2 cells (Figure 2a and b). Furthermore, we used RNA interference to downregulate the expression of KIAA1199 in LX-2 cells. Compared to the control siRNA, the expression of α-SMA and CoL1A1 were significantly decreased in LX-2 cells transfected with KIAA1199 siRNAs. Moreover, KIAA1199 siRNAs inhibited the elevation of α-SMA and CoL1A1 induced by TGF-β (Figure 2c and d). These results indicated that KIAA1199 was involved in HSC activation.

### 3.3 KIAA1199 knockdown inhibits proliferation and promotes apoptosis of HSCs

**Figure 3:**
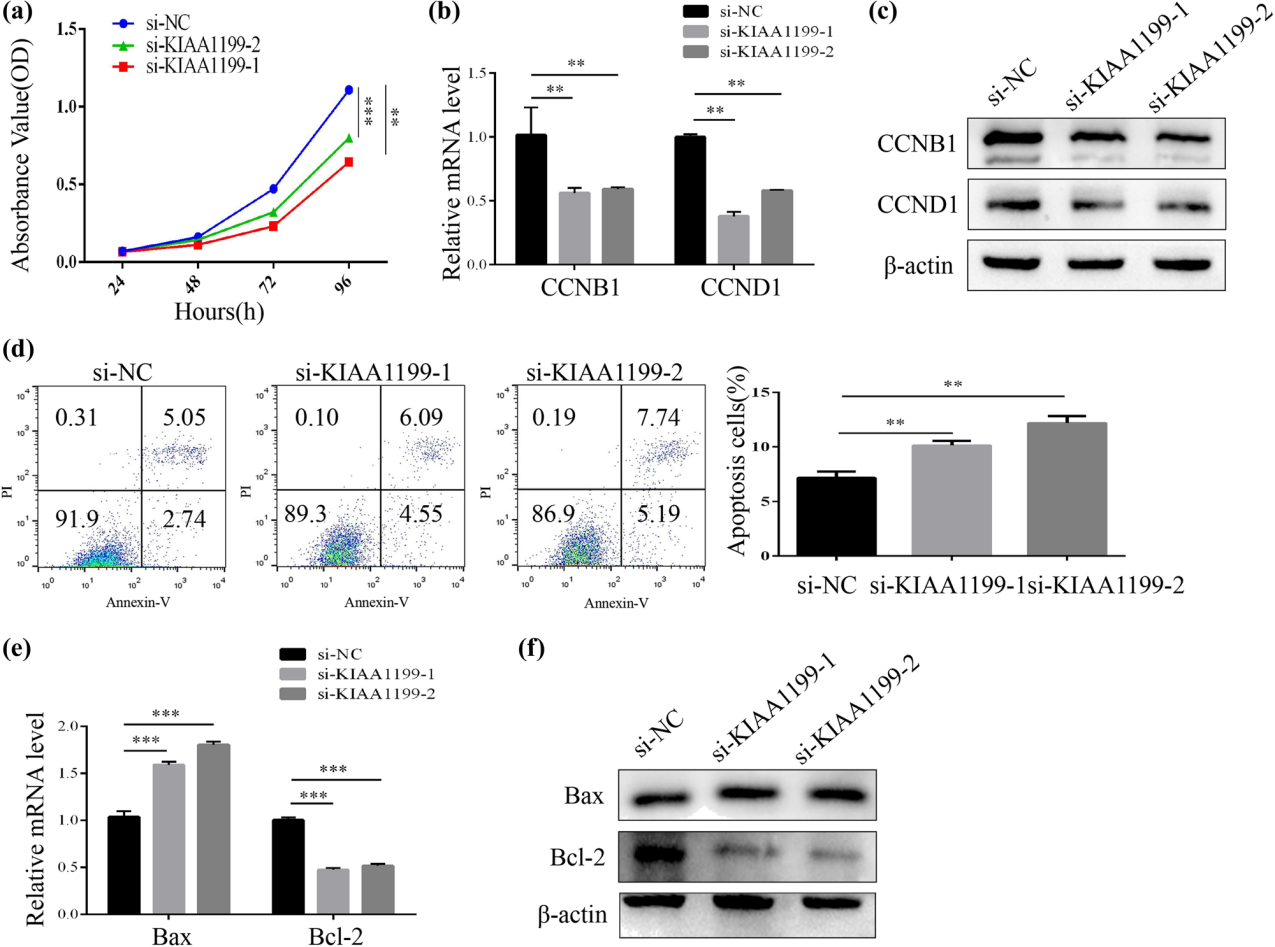
KIAA1199 knockdown inhibits proliferation and promotes apoptosis of HSCs. (a) The proliferation rates of LX-2 cells transfected with siRNAs (si NC or si KIAA1199) were determined by the CCK-8 assay. (b) RT-qPCR and (c) western blot analysis of cyclin B1(CCNB1) and cyclin D1(CCND1) expression in LX-2 cells transfected with siRNAs (si NC or si KIAA1199). (d) Flow cytometry shows the apoptosis of LX-2 cells transfected with siRNAs (si NC or si KIAA1199). (e) RT-qPCR and (f) western blot analysis of Bax and Bcl-2 expression in LX-2 cells transfected with siRNAs (si NC or si KIAA1199). β-Actin was used as a loading control. Each bar represents mean ± standard deviation of three separate experiments.^**^ *P* < 0.01 and ^***^ *P* < 0.001.

Next, we wondered whether KIAA1199 was associated with the proliferation and apoptosis of HSCs. The results of the CCK-8 assay demonstrated that KIAA1199 deficiency inhibited the proliferation of LX-2 cells (Figure 3a). To elucidate the underlying mechanism, we analyzed the expression of proliferation-related proteins. As shown, the expression of cyclin B1(CCNB1) and cyclin D1(CCND1) was decreased by KIAA1199 silencing (Figure 3b and c). In addition, the flow cytometry assay showed that the apoptosis rate of LX-2 cells transfected with KIAA1199 siRNAs was notably increased (Figure 3d). Compared to the control siRNA, pro-apoptotic protein Bax expression was remarkably increased, while anti-apoptotic protein Bcl-2 expression was decreased in KIAA1199 siRNAs-transfected LX-2 cells (Figure 3e and f). Collectively, these results suggested that downregulated KIAA1199 inhibited the proliferation and promoted apoptosis of HSCs.

### 3.4 KIAA1199 knockdown inhibited HSC migration and suppressed EMT

**Figure 4:**
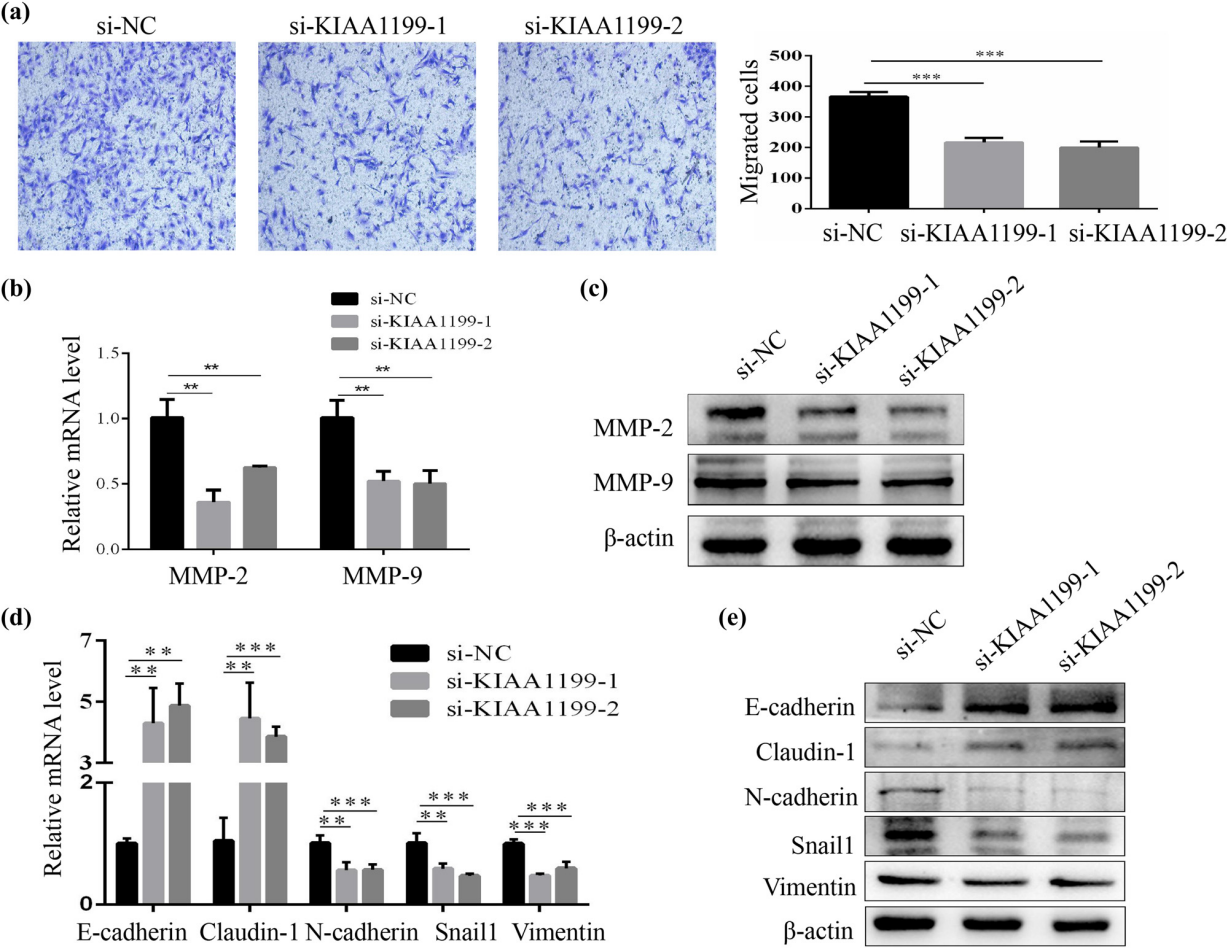
KIAA1199 knockdown inhibited HSC migration and suppressed EMT. (a) Cell migration in LX-2 cells (100×) was analyzed using the Transwell assay. (b) RT-qPCR and (c) western blot analysis of MMP-2 and MMP-9 expression in LX-2 cells transfected with siRNAs (si NC or si KIAA1199). (d) RT-qPCR and (e) western blot analysis of E-cadherin, Claudin-1, N-cadherin, Snail1, and vimentin expression in LX-2 cells transfected with siRNAs (si NC or si KIAA1199). β-Actin was used as a loading control. Each bar represents mean ± standard deviation of three separate experiments. ^**^ *P* < 0.01 and ^***^ *P* < 0.001.

We further investigated the role of KIAA1199 in the cell migration of HSCs. The Transwell assay demonstrated that LX-2 cells transfected with KIAA1199 siRNAs resulted in a decrease of the migration ability (Figure 4a). In addition, the expression of matrix metalloproteinases-2 (MMP-2) and MMP-9, which can promote cell migration [16], was remarkably decreased with the treatment of KIAA1199 siRNAs (Figure 4b and c). EMT is suggested to be one of the mechanisms in the activation and migration of HSCs [13]. We further explored the effect of KIAA1199 on EMT. The results demonstrated that the expression levels of the epithelial cell markers, E-cadherin and Claudin-1, were upregulated in LX-2 cells transfected with KIAA1199 siRNAs, while those of the mesenchymal cell markers such as N-cadherin, Snail1, and vimentin, were downregulated (Figure 4d and e). These results demonstrated that KIAA1199 knockdown inhibited HSC migration and suppressed EMT.

## 4 Discussion

Liver fibrosis is a progressive pathological disease in response to chronic liver injury [1]. Activation of HSCs is essential for the pathogenesis of liver fibrosis, which can transform into myofibroblasts and lead to excessive accumulation of ECM [3]. In this study, we focused on the role of KIAA1199 in liver fibrosis. The results revealed that KIAA1199 was upregulated in CCl_4_-induced mouse fibrotic liver tissues and TGF-β-stimulated LX-2 cells. Depletion of KIAA1199 inhibited activation, proliferation, and migration, while it promoted HSC apoptosis. Moreover, the KIAA1199 knockdown also suppressed the EMT. These findings suggested that KIAA1199 may be a therapeutic strategy for liver fibrosis.

KIAA1199, as a protein of emerging interest, is overexpressed in various cancers [6,7]. A recent study demonstrated that KIAA1199 induced a fibrosis-like process in osteoarthritic chondrocytes [14]. Our study found that KIAA1199 was overexpressed in the liver fibrosis animal model. As known, transforming growth factor-β (TGF-β) plays a pivotal role in liver fibrosis by regulating HSC activation and their transformation to myofibroblasts [15]. Consistent with a previous study [17], our study found that TGF-β treatment increased the expression of α-SMA and COL1A1 in LX-2 cells. Moreover, KIAA1199 expression was also upregulated by TGF-β stimulation. To elucidate the effect of KIAA1199 on HSC activation in-depth, we transiently downregulated KIAA1199 expression in LX-2 cells by RNA interference (RNAi). The results showed that KIAA1199 knockdown reduced the expression of α-SMA and COL1A1 and inhibited TGF-β-induced HSC activation, which indicated that KIAA1199 was involved in HSC activation.

In the pathological progression of liver fibrosis, the proliferation of activated HSCs amplifies the fibrotic response while the apoptosis of activated HSCs is thought to alleviate or reverse liver fibrosis [18,19]. Accumulating evidence has shown that KIAA1199 can regulate cell growth, migration, and invasion [8]. We observed that silencing KIAA1199 significantly suppressed the proliferation of LX-2 cells as indicated by the CCK8 assay, accompanied by decreased expression of two proliferation-related proteins, CCNB1 and CCND1. The flow cytometry assay showed that the apoptosis rate of KIAA1199 siRNA transfected LX-2 cells was significantly higher than that of control cells. Moreover, the level of the anti-apoptotic factor Bcl-2 in KIAA1199-siRNA transfected LX-2 cells was decreased, and the expression of the pro-apoptotic factor Bax was increased, indicating that KIAA1199 knockdown can promote the apoptosis of LX-2 cells. Taken together, KIAA1199 also participated in the proliferation and apoptosis of HSCs.

Recent studies have confirmed that the migration of activated HSCs is also important in the development of liver fibrosis [2,19]. Matrix metalloproteinases (MMPs), particularly MMP-2 and MMP-9, are upregulated in activated HSCs, which are pivotal to cell migration and responsible for liver fibrosis [20]. In this study, we found that silencing KIAA1199 inhibited LX-2 cell migration, which was accompanied by decreased MMP-2 and MMP-9 expression. Emerging evidence indicates that MMPs are related to the induction of EMT [21,22]. Moreover, KIAA1199 promotes gastric cancer cell migration and invasion by MMP-mediated EMT progression [10]. We further analyzed the effect of KIAA1199 on EMT in LX-2 cells. The results showed that silencing of KIAA1199 significantly increased the expression of epithelial biomarkers (E-cadherin, claudin-1) and decreased the expression of mesenchymal markers (N-cadherin, vimentin, Snail1) in LX-2 cells. Therefore, KIAA1199 knockdown inhibited EMT and HSC migration.

In conclusion, our study demonstrated that repression of KIAA1199 inhibited activation, proliferation, and migration of HSCs and promoted apoptosis of HSCs *in vitro*. Further studies should be carried out to clarify the role and molecular mechanism of KIAA1199 in liver fibrosis *in vivo*.
